# COVID-19 vaccine acceptance among pregnant women: a hospital-based cross-sectional study in Sudan

**DOI:** 10.3389/fpubh.2023.1221788

**Published:** 2023-07-17

**Authors:** Saeed M. Omar, Osama S. Osman, Rehana Khalil, Osama Al-Wutayd, Ishag Adam

**Affiliations:** ^1^Faculty of Medicine, Gadarif University, Gadarif, Sudan; ^2^Department of Family and Community Medicine, Unaizah College of Medicine and Medical Sciences, Qassim University, Unaizah, Saudi Arabia; ^3^Department of Obstetrics and Gynecology, Unaizah College of Medicine and Medical Sciences, Qassim University, Unaizah, Saudi Arabia

**Keywords:** COVID-19 vaccine, pregnancy, COVID-19, acceptance, Sudan

## Abstract

**Introduction:**

Pregnancy increases the risk of developing a severe illness due to COVID-19 infection. To the best of our knowledge, no previous study has been conducted on COVID-19 vaccine acceptance among pregnant women in Sudan. Hence, this study aimed to determine COVID-19 vaccination acceptance and its predictors among pregnant women.

**Methods:**

A cross-sectional study was conducted among 623 pregnant women attending Gadarif maternity hospital in eastern Sudan through a structured questionnaire. Data were obtained on sociodemographic characteristics, obstetric and health-related characteristics, COVID-19 infection, and vaccination-related information, as well as beliefs about and acceptance of COVID-19 vaccination.

**Results:**

COVID-19 vaccine acceptance among the pregnant women was 2.7%. The vaccine acceptance was higher if their husband’s education was secondary school or higher [adjusted odds ratio [AOR] 4.30, 95% confidence interval (CI) 1.11–16.65, *p* = 0.035] and discussion of COVID-19 vaccine with the pregnant women by a health care professional in the hospital (AOR 5.46, 95% CI 1.94–15.35, *p* < 0.001). The most common reasons for resistance to the vaccine were concerns about the side effects of the vaccine for the mother and her baby.

**Conclusion:**

Acceptance of the COVID-19 vaccination among the pregnant women was very low. Discussions with pregnant women and their husbands by health care professionals regarding the safety of COVID-19 vaccine for the mother and her baby are highly recommended.

## 1. Introduction

COVID-19 infections have been a major public health event since 2019 (1). The novel coronavirus has infected millions of people, disrupted health care systems, and caused global lockdowns (2). During the pandemic, the consistent use of face masks and maintenance of social distancing were identified as key preventive measures until mass vaccinations were initiated (3). Worldwide, once nations administered the vaccine, it substantially changed the course of the pandemic and saved millions of lives (4). Evidence has shown that pregnant women are likelier to become infected by SARS-CoV-2 (5). Pregnant women with COVID-19 infections are also at an elevated risk of severe illness and mortality (6, 7). Previous findings have shown that pregnant women hospitalized due to COVID-19 are likelier to be admitted to the intensive care unit (ICU), to require invasive ventilation, and to experience death compared with non-pregnant women with COVID-19 (8–11). Additionally, pregnant women with COVID-19 were found to have a higher risk of unfavorable birth outcomes, including stillbirth, preterm birth, cesarean delivery, and high rates of neonatal ICU admissions, compared with pregnant women without COVID-19 (12, 13). Limited evidence of vertical transmission of SARS-CoV-2 also exists (14). COVID-19 vaccination has been found to be the most efficient and effective way to prevent the transmission of SARS-CoV-2 to pregnant women and fetuses, as well as serious illness or other consequences, by producing immune responses during pregnancy (15, 16). The vaccine also protects the fetus or neonate against COVID-19 infection through the passive transplacental transfer of antibodies from the vaccinated mother during pregnancy (17). Thus, the Centers for Disease Control and Prevention, the Society for Maternal-Fetal Medicine, and the American College of Obstetricians and Gynecologists have strongly recommended that pregnant women be vaccinated to prevent maternal and fetal morbidity and mortality (18–20).

Low vaccine uptake has become a growing challenge worldwide (21–24). According to the World Health Organization (WHO), vaccine hesitancy was one of the top 10 health threats worldwide before the outbreak of the COVID-19 pandemic in 2019 (25). A previous literature review showed varying results for global vaccine acceptance during pregnancy (26).

Sudan was the first country in the Middle East and North Africa to obtain the COVID-19 vaccine through the Vaccines Global Access (COVAX) facility through the GAVI vaccine alliance, the Coalition for Epidemic Preparedness Innovations, the WHO, and UNICEF, a key delivery partner, in March 2021 (4, 27). By the end of 2021, it was anticipated that Sudan would obtain a 17 million doses from the COVAX facility, which would cover 20% of the populace (27). Furthermore, it was projected that in excess of 38 million doses would be secured to encompass at the minimum 45% of the Sudanese demographic prior to the realization of herd immunity (27, 28). Sudan administered the COVID-19 vaccine in three phases. Frontline health care workers were vaccinated in the first phase (28). In the second phase, people over 45 years of age with chronic medical conditions, school staff and teachers, and high-risk people who could not avoid exposure to COVID-19 due to the nature of their jobs were vaccinated (28, 29). In the third phase, pregnant women, lactating mothers, children, adolescents up to 16 years, and 80 people aged 16–45 years were vaccinated (29). In phase 3 of the vaccination campaign, there was a moderate level of vaccine availability to cater to this targeted population subset, ranging from 21 to 50% (28, 29). The financing of the third phase of COVID-19 vaccination necessitated the Sudanese government’s willingness to allocate funds toward the initiative amid the ongoing economic crisis (27). Internal funding and support for the health care system in Sudan have been lacking, which has been compounded by the long-standing economic sanctions in effect for two decades until 2017 (27, 28). To achieve the objective of vaccinating 50% of the population, the Sudanese government had to collaborate with external organizations to obtain funding and assistance in enhancing access to vaccines among citizens. Failure to do so would exacerbate the COVID-19 situation in the country (28).

The current fertility rate in Sudan is 4.4 births per woman (30). Previous studies have revealed varying rates of COVID-19 vaccine acceptance among pregnant women (31, 32). The acceptance rate for the COVID-19 vaccine among the Sudanese population has been low (33). However, no previous study has been conducted on acceptance of the COVID-19 vaccine by pregnant women in Sudan. Hence, this study aimed to determine COVID-19 vaccination acceptance and its predictors among pregnant women.

## 2. Materials and methods

### 2.1. Study design and setting

This observational cross-sectional study was conducted among pregnant women aged 18–44 years who attended the Gadarif Maternity Hospital in eastern Sudan from July through September 2022. Gadarif, one of Sudan’s 18 states, has an area of 75,263 km^2^ and an estimated population of 1,827,181 (34, 35). This semi-desert tropical region is located in southeastern Sudan between latitudes 14 and 16 north and longitudes 35 and 36 east (34). Its main sources of income are farming, trading, and animal breeding (35, 36).

### 2.2. Data collection

#### 2.2.1. Questionnaire

Face to face interviews was conducted using questionnaire which was framed according to previously published research and the objectives of this study (37–42). Three experts reviewed and approved the content validity of the questionnaire. Before the main study, the questionnaire was pilot tested on 20 respondents. The final version of the questionnaire was revised and approved by all the authors.

#### 2.2.2. Study variables

##### 2.2.2.1. Dependent variable

The dependent variable was assessed by the question “Do you intend to receive the COVID-19 vaccine?” (yes/no).

##### 2.2.2.2. Independent variables

- Sociodemographic information, including age, place of residence (i.e., urban or rural), level of education (lower than secondary school, secondary school, or higher), level of education of the pregnant woman’s husband (lower than secondary school, secondary school or higher), working status over the last 12 months (housewife or employed), residing with someone older than 60 years (yes/no).
- Obstetric and health-related characteristics, including parity, history of miscarriage (yes/no), gestational age in weeks, and history of comorbidity (i.e., diabetes mellitus, hypertension, thyroid disease, other, or none).
- COVID-19 infection and vaccination-related information, including history of COVID-19 infection before and during the current pregnancy; history of COVID-19 infection in the pregnant woman’s husband (yes/no); worry of COVID-19 infection during pregnancy (yes, no); and COVID-19 vaccine is safe and effective (yes, no); receipt of COVID-19 vaccine by the pregnant woman’s husband (yes or no); COVID-19 vaccine discussed with the pregnant woman by any hospital staff (i.e., midwife, doctor, unsure, or not yet discussed).
- Beliefs about and acceptance of COVID-19 vaccination, including the pregnant woman’s worry of side effects of the COVID-19 vaccination for herself (strongly agree, agree, disagree, or strongly disagree); the pregnant woman’s worry of side effects of COVID-19 vaccination on her baby (strongly agree, agree, disagree, or strongly disagree); inadequate information about the safety of the COVID-19 vaccine during pregnancy (strongly agree, agree, disagree, or strongly disagree); unsuitable times for scheduling an appointment to receive the COVID-19 vaccine during pregnancy (strongly agree, agree, disagree, or strongly disagree); the belief that COVID-19 in Gadarif is decreasing, and there is no need to be vaccinated against it (strongly agree, agree, disagree, or strongly disagree).

#### 2.2.3. Sample size calculation

**Figure 1:**
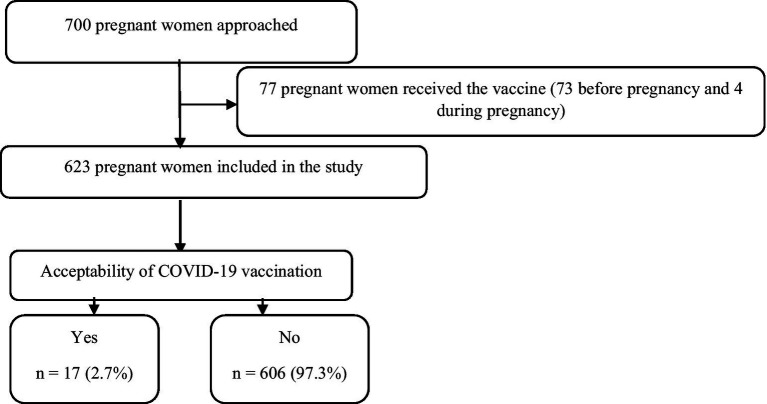
Flow diagram of the study participants.

The sample size was calculated using OpenEpi software. The minimum sample size was 500 participants (95% confidence interval [CI], 80% statistical power, and 5% type I error). The rate of COVID-19 vaccination acceptance among pregnant women was assumed to be 50%, based on a lack of related studies in Sudan to attain the maximum sample size (30% expected incomplete and missing responses; Figure 1).

### 2.3. Ethics approval

The ethics committee of Gadarif University approved the current study (reference number 15,06,2022). The purpose of this study was thoroughly explained to the participants, their informed consent was obtained, and their information was treated confidentially by the researchers. All study procedures were performed in accordance with the ethical standards of the institutional and/or national research committee and with the 1964 Helsinki Declaration and its later amendments.

### 2.4. Data analysis

The data were entered into an Excel sheet and checked to exclude duplicate answers, after which they were exported to STATA version 16 for statistical analysis. The categorized data are expressed as frequencies and percentage (%). The continuous data were not normally distributed and are expressed as medians and interquartile ranges (IQRs). Bivariate and multivariable analyses were conducted to determine associations between the independent variables (i.e., potential predictors) and the dependent variable (i.e., vaccine acceptance). Multiple logistic regressions included potential predictors with a value of *p* of <0.25 in the simple logistic regression. A value of *p* of <0.05 was considered robust evidence against the null hypothesis.

## 3. Results

A total of 623 participants were included in this study. The median (IQR) age was 28 (24–32) years, the median (IQR) gestational age was 32 (27–38) weeks, and the median (IQR) parity was 3 (2–5). Two-thirds (66.4%) of the participants lived in rural areas. The numbers and proportions of the pregnant women and their husbands who had secondary school or higher-level education were 369 (59.2%) and 311 (49.9%), respectively. The majority (570, 91.5%) were housewife, participants with people over 60 years in their households numbered 434 (69.7%), and 27 (4.3%) of the pregnant women had comorbidities (Table 1). Only 17 (2.7%) of the pregnant women were willing to receive the COVID-19 vaccine, none of whom had a history of COVID-19 infection (Table 2). The results of the bivariate analysis showed that the pregnant women were likelier to receive the COVID-19 vaccine if their husbands had a secondary school education or higher (COR 4.86, 95% CI 1.38–17.07, *p* = 0.014), were employed (COR 3.49, 95% CI 1.09–11.14, *p* = 0.034), if their husbands had a history of COVID-19 infection (COR 9.97, 95% CI 1.94–50.97, *p* = 0.006), if they themselves believed that the COVID-19 vaccine was safe (COR 6.59, 95% CI 2.49–17.63, *p* < 0.001) and effective (COR 6.14, 95% CI 2.26–16.71, *p* < 0.001), and if hospital staff had discussed the COVID-19 vaccine with them (COR 5.46, 95% CI 1.94–15.35, *p* < 0.001; Table 3).

**Table 1:** Sociodemographic and health related data of the study participants (*n* = 623).

	Median	Interquartile range
Age, years	28	24–32
Gestational age, weeks	32	27–38
Parity	3	2–5
	Frequency	Percentage
Residence		
Urban	209	33.6
Rural	414	66.4
Education level		
Lower than secondary school	254	40.8
Secondary school or higher	369	59.2
Husband’s education level		
Lower than secondary school	312	50.1
Secondary school or higher	311	49.9
Occupation		
Housewife	570	91.5
Employed	53	8.5
People in the household over 60 years		
Yes	434	69.7
No	189	30.3
Comorbidity		
Yes	27	4.3
No	596	95.7

**Table 2:** Comparing different variables between women who accepted COVID-19 vaccination and women who did not.

Variable		Acceptability of COVID-19 vaccination among pregnant women	
		Yes (number = 17)	No (number = 606)
		Median (interquartile range) of	
Age		26 (25–31)	28 (24–32)
Gestational week		31 (21–36)	32 (28–38)
Parity		3 (2–5)	3 (2–5)
		Number (percentage) of	
Residence	Urban	8 (3.8)	201 (96.2)
	Rural	9 (2.2)	405 (97.8)
Education level	Secondary school or higher	13 (3.5)	356 (96.5)
	Lower than secondary school	4 (1.6)	250 (98.4)
Husband’s education	Secondary school or higher	14 (4.5)	297 (95.5)
	Lower than secondary school	3 (1)	309 (99)
Occupation	Employed	4 (7.6)	49 (92.4)
	Housewife	13 (2.3)	557 (97.7)
People in the household over 60 years	Yes	12 (2.8)	422 (97.2)
	No	5 (2.7)	184 (97.3)
History of miscarriage	Yes	4 (2.4)	160 (97.6)
	No	13 (2.8)	446 (97.2)
Comorbidity	Yes	2 (7.4)	25 (92.6)
	No	15 (2.5)	581 (97.5)
History of COVID-19 infection in my husband	Yes	2 (20)	8 (80)
	No	15 (2.5)	598 (97.6)
Worry about COVID-19 infection	Yes	9 (4.6)	185 (95.4)
	No	8 (1.9)	421 (98.1)
COVID-19 vaccine is safe	Yes	8 (10)	72 (90)
	No	9 (1.7)	534 (98.3)
COVID-19 vaccine is effective	Yes	7 (10.1)	62 (89.9)
	No	10 (1.8)	544 (98.2)
COVID-19 vaccine received by my husband	Yes	1 (11.1)	8 (88.9)
	No	16 (2.6)	598 (97.4)
COVID-19 vaccine discussed with you by any staff in the hospital	Yes	6 (9.8)	55 (90.2)
	No	11 (2)	551 (98)

**Table 3:** Simple and multiple logistic regression analyses exploring the predictors of acceptability of COVID-19 vaccination among pregnant women.

Variable		Bivariate analysis	*p* value	Multivariable analysis	*p* value
		COR (95% CI)		AOR (95% CI)	
Age, years		0.99 (0.90–1.09)	0.828		
Gestational, weeks		0.97 (0.92–1.02)	0.266		
Parity		0.94 (0.73–1.19)	0.588		
Residence	Urban	Reference			
	Rural	1.79 (0.68–4.71)	0.238	1.06 (0.33–3.41)	0.922
Mother’s education level	Secondary school or higher	Reference		Reference	
	Lower than secondary school	2.28 (0.74–7.08)	0.153	1.19 (0.32–4.44)	0.800
Husband’s education	Secondary school or higher	Reference		Reference	
	Lower than secondary school	4.86 (1.38–7.07)	0.014	4.30 (1.11–16.65)	0.035
Occupation	Employed	Reference		Reference	
	Housewife	3.49 (1.09–1.14)	0.034	1.47 (0.34–6.41)	0.605
People in the household over 60 years	Yes	Reference			
	No	1.05 (0.36–3.01)	0.933		
History of miscarriage	No	Reference			
	Yes	0.86 (0.28–2.67)	0.791		
Comorbidity	No	Reference			
	Yes	3.09 (0.67–14.29)	0.147	2.46 (0.43–14.14)	0.312
History of COVID-19 infection in my husband	No	Reference		Reference	
	Yes	9.97 (1.94–50.97)	0.006	2.04 (0.22–18.65)	0.527
Worry about COVID-19 infection	Yes	Reference		Reference	
	No	2.56 (0.97–6.74)	0.057	0.66 (0.17–2.57)	0.547
COVID-19 vaccine is safe	Yes	Reference		Reference	
	No	6.59 (2.47–17.63)	<0.001	2.42 (0.45–13.05)	0.303
COVID-19 vaccine is effective	Yes	Reference			
	No	6.14 (2.26–16.71)	<0.001	2.63 (0.48–14.29)	0.264
COVID-19 vaccine received by my husband	Yes	Reference		Reference	
	No	4.67 (0.55–39.60)	0.157	2.99 (0.23–39.13)	0.405
COVID-19 vaccine discussed with you by any staff in the hospital	Yes	Reference		Reference	
	No	5.46 (1.94–15.35)	0.001	3.89 (1.16–13.07)	0.027

Adjusted for parity.

COR, crude odds ratio; CI, confidence interval; and AOR, adjusted odds ratio.

Goodness of fit was evaluated by area under ROC curve which was 0.80 in the model.

The results of the multivariable analysis showed that the variables that remained statistically significant predictors were as follows: husband’s education equal to secondary school or higher (adjusted odds ratio [AOR] 4.30, 95% CI 1.11–16.65 *p* = 0.035); and hospital staff discussed the COVID-19 vaccine with the pregnant women (COR 5.46, 95% CI 1.94–15.35, *p* < 0.001; Goodness of fit was evaluated by area under ROC curve which was 0.80 in the model; Table 3). Among the participants, the most frequent reason for not accepting the COVID-19 vaccination was concern about the side effects of the vaccine on the mother and her baby. The second most frequent reason was inadequate information about the safety of the vaccine, followed by the participants’ inability to find a suitable time to schedule an appointment for the vaccine, and the belief that the rate of COVID-19 infection was decreasing in the city of their residence (Table 4).

**Table 4:** Reasons for not accepting COVID-19 vaccination (*n* = 606).

Item	Strongly agree *n* (%)	Agree *n* (%)	Disagree *n* (%)	Strongly disagree *n* (%)
I am worried about the side effects of the COVID-19 vaccination for myself.	233 (38.5)	346 (57.1)	22 (3.6)	5 (0.8)
I am worried about the side effects of the COVID-19 vaccination for my baby.	237 (39.1)	336 (55.5)	30 (5)	3 (0.5)
I cannot find enough adequate information on the safety of the COVID-19 vaccine during pregnancy.	257 (42.4)	300 (49.5)	44 (7.3)	5 (0.8)
I cannot find any suitable time to schedule an appointment for my COVID-19 vaccine.	211 (36.5)	271 (44.7)	111 (18.3)	3 (0.5)
I believe that the decreased rates of COVID-19 in Gadarif mean I do not need to be vaccinated.	190 (31.4)	270 (44.5)	125 (20.6)	21 (3.5)

## 4. Discussion

To the best of our knowledge, our study is the first to examine COVID-19 vaccine acceptance by pregnant women in Sudan. The findings showed a rate of COVID-19 vaccine acceptance in pregnant women of 2.7%, which is lower than the acceptance rate among African countries like Ethiopia (70.7%), South Africa (63.3%), and Cameroon (31%) (43–45). A systematic review and meta-analysis reported wide variations in countries around the globe for COVID-19 vaccine uptake among pregnant women, ranging from 7.0 to 68.7% (46). Contemporary research studies have reported an acceptance rate below 40% among pregnant women in India (18%), Singapore (30.3%), Ireland (38%), Turkey (37%), France (29.5%), and Japan (13.4%) (37, 47–51). On the other hand, China (77.4%), Thailand (60.8%), Saudi Arabia (68%), Qatar (75%), the United Kingdom (62.1%), Czechia (76.6%), Italy (84.5%), and the United States (76.2%) were reported to have more than a 60% acceptance rate among pregnant women (10, 32, 41, 42, 52–55).

The current study showed the lowest acceptance rate (2.7%) of COVID-19 vaccine uptake among pregnant women worldwide. The Africa CDC have reported a fully vaccinated rate for Sudan of 40.2% (56). However, our research indicates a notable lack of vaccination acceptance among pregnant women, which could result in a failure to reach the targeted phase 3 vaccination range of 21–50% in Sudan. This reluctance to receive a COVID-19 vaccination can be efficiently resolved by addressing the specific reasons that have been identified in our study. The primary concerns most commonly expressed by expectant mothers pertained to the safety of both their own health and that of their fetuses, thus corroborating the conclusions drawn from numerous antecedent studies (31, 37, 57–60). Additionally, a lack of sufficient information regarding the vaccine’s safety, as well as the inability to schedule a suitable time for the COVID-19 vaccination appointment during pregnancy, were cited as contributing factors in our research. The matter of vaccination trials or administration during pregnancy in Africa is shrouded in silence (61). The Pfizer-BioNTech vaccine, which has been approved, is not a live virus vaccination but rather an mRNA vaccine, and the Royal College of Obstetricians and Gynecologists has declared that there is insufficient evidence to support the routine use of COVID-19 vaccines for pregnant or breastfeeding women. Furthermore, women who plan to conceive within 3 months of receiving the first dose have been advised against getting vaccinated (62). The accelerated vaccine development process, which has employed modern technologies, has increasingly come under scrutiny (63). A survey conducted between August and December 2020, spanning 15 African countries, including Burkina Faso, Côte d’Ivoire, Democratic Republic of the Congo, Ethiopia, Gabon, Kenya, Malawi, Morocco, Niger, Nigeria, Senegal, South Africa, Sudan, Tunisia, and Uganda, polled over 15,000 adults aged 18 years and above. The Africa CDC collaborated with the London School of Hygiene & Tropical Medicine to conduct the survey, which revealed that a considerable majority (an average of 79%) of respondents in Africa would get vaccinated against COVID-19 if it was deemed safe and effective (64). Despite the absence of unequivocal proof regarding the timing and adverse effects of COVID-19 vaccination in pregnant women, no previous observational study or large case series has evinced any deleterious impact of the COVID-19 vaccine on the health of pregnant women or their neonates (65–71).

The findings of the present study showed an association between pregnant women whose husband’s education was equal to or higher than secondary level and a higher rate of COVID-19 vaccine acceptance. This can be rationalized by the indispensable role played by husbands in the process of making informed decisions pertaining to the administration of vaccines for women (72). This finding is similar to a study by Tefera et al. (73), who attributed the association to the ability of educated husbands to read and communicate to their wives news and social media messages related to the seriousness of COVID-19 infection and the positive effects of the vaccine. In Sudan, gender roles are characterized by a strong patriarchal influence, with rigid definitions in place. The male population is predominantly perceived as the primary breadwinners and decision-makers, while their female counterparts are predominantly viewed as homemakers. It is expected that husbands take on the economic responsibility of providing for their wives and children throughout their lifetimes (74).

Another important finding of our study was that the vaccination likelihood of pregnant women with whom any hospital staff discussed the potential benefits of the COVID-19 vaccine was higher compared with those who did not have that experience. This finding is consistent with the findings of recent studies by Riad et al. (10) in Czechia and Nganga et al. (75) in Kenya, which showed that the COVID-19 vaccine was likelier to be accepted by pregnant women if it was recommended by health care professionals (HCPs) and it was made accessible to them. The same findings were endorsed in a literature review by Wilson et al. (26). Therefore, it is highly recommended that HCPs be educated about evidence-based recommendations for maternal vaccinations during pregnancy to enable them to address vaccine-hesitant pregnant women (76, 77). The present study had the following limitations. Causal relationships could not be established because of the inherent limitations of the cross-sectional study design, the time factor in conducting the study during the COVID-19 pandemic produced limitations for properly validating the questionnaires, and the validation consisted only of expert judgment and a pilot study on 20 mothers, which is inadequate for proper psychometric validation of the tool.

## 5. Conclusion

Acceptance of the COVID-19 vaccination among the pregnant women in this study was extremely low. Discussions with pregnant women and their husbands by an HCP regarding the safety of the COVID-19 vaccine for both mother and baby are highly recommended. We also recommend that future vaccination campaigns focus on enhancing HCPs’ communication skills to ensure the engagement of pregnant women and promote vaccination uptake during pregnancy. The findings of our study could provide guidance for health care providers and policymakers in designing effective strategies to improve vaccine uptake during pregnancy in eastern Sudan.

## Data availability statement

The raw data supporting the conclusions of this article will be made available by the authors, without undue reservation.

## Ethics statement

The ethics committee of Gadarif University was approved the current study (reference number 15,06,2022). The patients/participants provided their written informed consent to participate in this study.

## Author contributions

IA and OA-W conceived and designed the study. OO and SO prepared the manuscript. OO, IA, and SO supervised the study, and OA-W, RK, and IA contributed to the paper editing. All authors contributed to the article and approved the submitted version.

## Conflict of interest

The authors declare that the research was conducted in the absence of any commercial or financial relationships that could be construed as a potential conflict of interest.

## Publisher’s note

All claims expressed in this article are solely those of the authors and do not necessarily represent those of their affiliated organizations, or those of the publisher, the editors and the reviewers. Any product that may be evaluated in this article, or claim that may be made by its manufacturer, is not guaranteed or endorsed by the publisher.
